# DMPy: a Python package for automated mathematical model construction of large-scale metabolic systems

**DOI:** 10.1186/s12918-018-0584-8

**Published:** 2018-06-19

**Authors:** Robert W. Smith, Rik P. van Rosmalen, Vitor A. P. Martins dos Santos, Christian Fleck

**Affiliations:** 1 Laboratory of Systems & Synthetic Biology, Wageningen UR, Stippeneng 4, Wageningen, 6708WE The Netherlands; 2 LifeGlimmer GmbH, Markelstrasse 38, Berlin, 12163 Germany

**Keywords:** Genome-scale, Constraint-based metabolic models, Automated data collection, Dynamic mathematical model, Parameter optimisation

## Abstract

**Background:**

Models of metabolism are often used in biotechnology and pharmaceutical research to identify drug targets or increase the direct production of valuable compounds. Due to the complexity of large metabolic systems, a number of conclusions have been drawn using mathematical methods with simplifying assumptions. For example, constraint-based models describe changes of internal concentrations that occur much quicker than alterations in cell physiology. Thus, metabolite concentrations and reaction fluxes are fixed to constant values. This greatly reduces the mathematical complexity, while providing a reasonably good description of the system in steady state. However, without a large number of constraints, many different flux sets can describe the optimal model and we obtain no information on how metabolite levels dynamically change. Thus, to accurately determine what is taking place within the cell, finer quality data and more detailed models need to be constructed.

**Results:**

In this paper we present a computational framework, DMPy, that uses a network scheme as input to automatically search for kinetic rates and produce a mathematical model that describes temporal changes of metabolite fluxes. The parameter search utilises several online databases to find measured reaction parameters. From this, we take advantage of previous modelling efforts, such as Parameter Balancing, to produce an initial mathematical model of a metabolic pathway. We analyse the effect of parameter uncertainty on model dynamics and test how recent flux-based model reduction techniques alter system properties. To our knowledge this is the first time such analysis has been performed on large models of metabolism. Our results highlight that good estimates of at least 80% of the reaction rates are required to accurately model metabolic systems. Furthermore, reducing the size of the model by grouping reactions together based on fluxes alters the resulting system dynamics.

**Conclusion:**

The presented pipeline automates the modelling process for large metabolic networks. From this, users can simulate their pathway of interest and obtain a better understanding of how altering conditions influences cellular dynamics. By testing the effects of different parameterisations we are also able to provide suggestions to help construct more accurate models of complete metabolic systems in the future.

**Electronic supplementary material:**

The online version of this article (10.1186/s12918-018-0584-8) contains supplementary material, which is available to authorized users.

## Background

Quantitative modelling of metabolic networks has helped design and improve the production of compounds relevant for the bio-industrial and pharmaceutical sectors [1, 2]. Many of the favoured methods to model large metabolic networks relate system inputs (e.g. growth conditions) to phenotypic outputs (for example, growth rate or compound secretion) using directed graphs, i.e. internal dynamics are not directly considered [3, 4]. However, to obtain a more detailed understanding of how system perturbations or alterations influence metabolic networks and, hence, the observed and predicted phenotypes, it is desirable to have a method of constructing consistent kinetic models of large-scale metabolism. Here, we present a computational pipeline that brings together a range of recently published methods to convert a genome-scale reaction network into a detailed mathematical model, usable to study and predict metabolic functions [5].

In mathematical terms, large-scale metabolic networks are represented by

1 $$ \frac{d\mathbf{x}}{dt} = \mathbf{S}\cdot\mathbf{v}\left(\mathbf{x},\mathbf{k}\right),  $$

where **x** is the vector of metabolite concentrations, **S** is the stoichiometry matrix of the system detailing how one metabolite converts into another, and **v**(**x**,**k**) is the flux vector that describes the rate of metabolite conversion. Notably, the fluxes will generally depend on the concentrations of metabolites in the network and the parameters, or kinetic rates, **k**. Methods to solve Eq. 1 have approached the problem from two directions. First, by using the quasi-steady-state assumption, the metabolite concentrations are believed to be constant relative to the time taken for a cell to grow [4, 6]. This method requires data such as growth and production rates of the species under study [7, 8]. Furthermore, gene expression and essentiality data, or metabolic flux measurements (for example from ^13^C-labelling experiments), can be used to further constrain and validate the model. Second, by using mathematical functions (based on generalised Michaelis-Menten kinetics) to describe **v**(**x**,**k**) with measured or estimated values of **k** and approximating the system solution numerically [9–11]. The model simulations can then be compared to measured time-series profiles of metabolite concentrations. We shall briefly review both approaches here.

### Constraint based metabolic models

The result of the quasi-steady-state assumption simplifies Eq. 1 to

2 $$ \mathbf{S}\cdot\mathbf{v} = \mathbf{0},  $$

which is a set of linear equations that can be solved for an optimal vector **v** when the system is optimized to, for example, maximize biomass production (although there are a variety of other functions, as shown in [7]). This method is commonly referred to as Flux Balance Analysis (FBA) [4, 6, 12]. Notable examples highlighting the utility of FBA approaches can be found in [3, 13–15] whilst [16] have recently used FBA to obtain a genome-scale model of the human metabolic network, which has seen use in applications such as the discovery of anticancer drugs [17, 18]. Over recent years, a number of extensions to the FBA method have been developed. However, systematic analysis of these methods has shown that there is not one single best-performing method that provides a reasonable match between simulated and measured reaction fluxes [2, 7, 8].

One notable development of FBA is dynamic FBA (dFBA) that aims to match time-dependent changes in system outputs with internal flux dynamics [19–21]. In principle, dFBA is a set of FBA computations conducted independently at several time-points. This results in a set of dynamically changing optimal flux vectors that cover the analysed time-period. However, such an approach can be problematic as the solution of FBA is often non-unique, i.e. there are multiple optimal flux vectors that can solve Eq. 2 for a given set of objective functions [22, 23]. Consequently, discontinuities in the flux vector can be observed when **v** is plotted against time, which is suggestive of a jump from one optimal flux vector to another [20, 21]. One method of solving this issue is to constrain the flux optimisation such that the time-dependent change from one flux vector to another is not allowed to be large, thus limiting the search space for the optimisation routine [19, 20, 24]. The addition of constraints such as these enforces the reaction fluxes of a network to change continuously through time, in a similar manner to trajectories obtained from Eq. 1, but it is not clear how the trajectories depend on reaction rates or concentrations.

### Dynamic metabolic models

In the second approach to solving Eq. 1, one has the difficulty that the mathematical form of **v**(**x**,**k**) and the parameters **k** need to be approximated in the absence of appropriate and detailed datasets. Often the mathematical form of the fluxes **v**(**x**,**k**) are approximated using Michaelis-Menten kinetics [9, 11, 25, 26]. Notably, the use of Michaelis-Menten kinetics has been found to provide accurate results for large-scale metabolic models when compared to other frequently used rate laws and has been useful in analysing the robustness of dynamics in metabolic networks [25, 27, 28]. The generalised/reversible form of the Michaelis-Menten approximation for a reaction $\sum _{i=1}^{n}\alpha _{i}A_{i} \rightleftharpoons \sum _{j=1}^{m}\beta _{j}B_{j}$ reads

3 $$ {}v\left(\{\mathbf{a},\mathbf{b}\},\mathbf{k}\right) = u_{l}\frac{k^{cat+}\prod_{i}\left(a_{i}/k_{a_{i}}^{M}\right)^{\alpha_{i}}-k^{cat-}\prod_{j}\left(b_{j}/k^{M}_{b_{j}}\right)^{\beta_{j}}}{\prod_{i}\left(a_{i}/k^{M}_{a_{i}}\right)^{\alpha_{i}}+\prod_{j}\left(b_{j}/k^{M}_{b_{j}}\right)^{\beta_{j}}-1}  $$

with *u*_*l*_ representing the enzyme concentration for reaction *R*_*l*_ and $\mathbf {k} =\allowbreak \{k^{cat+},\allowbreak k^{cat-},\allowbreak k^{M}_{a},\allowbreak k^{M}_{b},\allowbreak \boldsymbol {\alpha },\allowbreak \boldsymbol {\beta }\}$, where *k*^*cat*^ are the catalytic rates, *k*^*M*^ are the Michaelis constants and *α*_*i*_ (or *β*_*j*_) the reaction stoichiometry associated with concentration *a*_*i*_ of reactant *A*_*i*_ (or *b*_*j*_ of product *B*_*j*_) [9]. What one should notice immediately is that, for even a simple reaction, a large number of kinetic rates need to be known or accurately estimated to ensure that Eq. 3 matches observed data (at least 4 rates for a reaction with a single reactant and product). Thus, whereas the pitfall of dFBA is that it requires a large number of system constraints to provide an accurate solution, the downside to kinetic approaches is that a vast amount of data is required to accurately parameterise models of large metabolic networks.

### Parameter balancing

Fortunately, an increasing number of experimentally-measured parameters required for the use of Michaelis-Menten approximations are becoming available in online databases (for example BRENDA [29], SABIO-RK [30] and eQuilibrator [31]). Parenthetically, even if only in vitro estimates of kinetic rates are available, in certain cases the relationship to their in vivo value has been shown [32]. Furthermore, the Parameter Balancing (referred to as PB from hereon) method has been developed to utilise Bayesian inference techniques and include constraints on thermodynamic relationships between different parameters [33, 34]. Thus, one could obtain either measured or realistic estimates for a number of the parameters required to construct a kinetic model and simulate changes in metabolite concentration. Examples of such steps can be found in [1, 11], whilst [28] have shown that relatively small models constructed with fluxes given by Eq. 3 together with measured or estimated parameter values provides better matches to data than other tested functions.

### Model reduction

The examples of [1, 11] show two different desired cases of modelling metabolism. In the first instance, [1] construct a detailed model describing the central metabolic pathways of *L. lactis* whilst, in the second, [11] produce a genome-scale model of yeast metabolism. In principle, the conclusions drawn from larger models should be consistent with those of smaller, more detailed systems and *vice versa* when created in the same species. Thus one important consideration is that of model reduction. Based on current methods, model reduction could occur at one of three stages: pruning unimportant reactions directly from the genome-scale network [35], grouping subsets of reactions into a single effective reaction on the subsystem of interest [36], or by assessing the kinetic rates of the system using time-scale separation arguments fixing a subset of system components to constant values [37]. Notably, the methods of [35, 36] require only the reaction network and no information about the dynamics of the pathway in order to reduce the system size.

### DMPy

Here, inspired by the workflows of [5, 11], we present an automated pipeline that can translate a (genome-scale) reaction network into a dynamic reaction equation model. Given the network as an input, our pipeline automatically integrates measured kinetic rates from different sources (both online databases and measured or estimated values) with the PB technique, optionally reduces model size and, finally, translates the network into ordinary differential equations using Eq. 3. Importantly, this method differs from other (semi-automatic) methods of constructing ODE models (such as CellNetAnalyzer and COPASI [38, 39]) as parameters are obtained from readily-available experimental measurements rather than including an optimisation step to time-series data that can prove very difficult for large-scale systems. By generating simulated data of both the *L. lactis* central metabolic pathway and randomly generated reaction networks, we go on to analyse the accuracy of our pipeline and determine how many kinetic rates must be measured experimentally to obtain an accurate model. We also show the effect of flux-based model reduction techniques on the resulting system dynamics, extensions to larger networks, and the utility of our pipeline by including compartmentalisation and regulation within metabolic pathways. The presented framework is intended to provide a first approximation of large-scale metabolic dynamics within which further details and computational methods can be added as more data and information come to light. In the Discussion we will highlight how our pipeline can be extended to improve the resulting models (by incorporating different model reduction and parameter optimisation strategies) if the appropriate datasets are available.

## Implementation and methods

### Pipeline

In this section we shall provide an overview of the computational workflow. For further implementation details and required inputs please refer to Additional file 1. All simulations and testing were performed using Python version 2.7 (Python Software Foundation, www.python.org) and MATLAB R2012b (MathWorks, Massachusetts, USA).

**Fig. 1:**
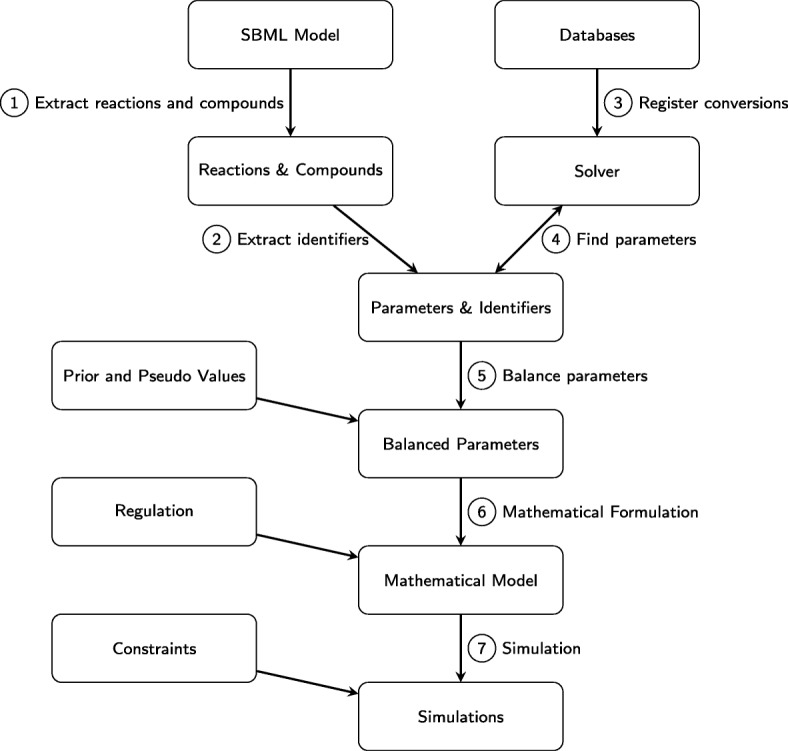
An overview of the computational framework. The pipeline consists of seven stages. The first of these (Steps 1-2) parses a genome-scale SBML model such that the reactions and components within the system are listed. At the same time, the list of databases to be searched is registered and the kinetic rates for each reaction in the metabolic network are found (Steps 3-4). Upon obtaining the kinetic rates, PB is used to obtain thermodynamically feasible parameter distributions (Step 5). Using these parameter values, the SBML model is then translated into a set of ordinary differential equations representing the dynamic changes in system components (Step 6). Finally, the parameterised model can be simulated and analysed (Step 7)

Figure 1 provides a pictorial overview of the pipeline and consists of three main parts. First, a genome-scale reaction network in SBML (Systems Biology Markup Language, www.sbml.org [40]) format is used as an input into DMPy. The model is parsed and relevant kinetic rates are found from a range of online databases (Steps 1-4, Fig. 1). Then, the obtained parameter estimates (both from online databases and experimental measurements) are used as an input into the PB software that generates a set of thermodynamically consistent kinetic rates (Step 5, Fig. 1). Finally, the kinetic rates are input into Michaelis-Menten functions with similar form to Eq. 3 such that the SBML reaction network can be translated into parameterised ordinary differential equations (Step 6, Fig. 1). The model can now be simulated and edited as required. To look at the effect of model reduction upon system dynamics, we have used flux-based model reduction techniques prior to inputting the genome-scale model into the pipeline [35]. In the Discussion we will explain the implication of doing this and consider alternative methods.

In the following we shall discuss each stage of the pipeline and its purpose.

#### Parameter gathering (steps 1-4, Fig. 1)

**Fig. 2:**
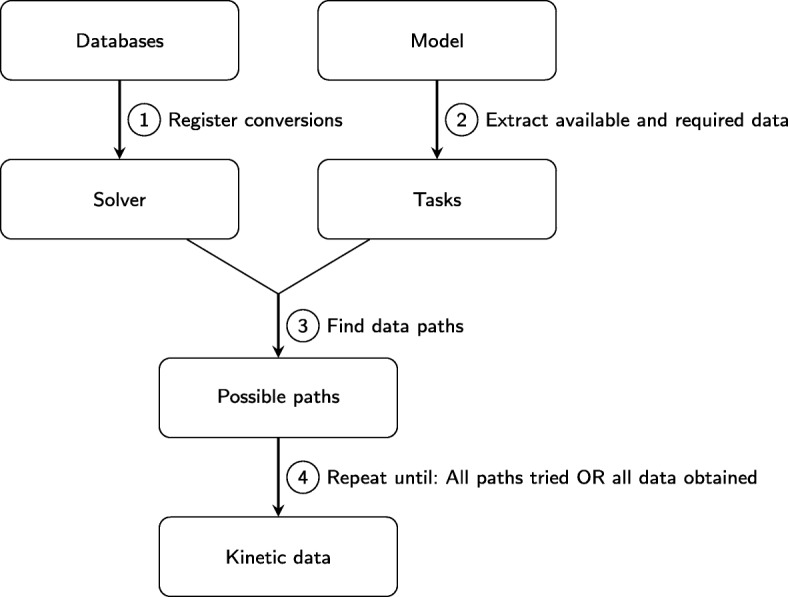
Automated search for parameter values. In order to automatically find all measured kinetic rates from online databases, a subroutine was constructed as part of our pipeline (see Additional file 1). Upon registering the databases to be searched and how their identifiers can be converted between each other (Step 1), all the reactions within the parsed genome-scale model are listed (Step 2). Then, the algorithm finds all possible paths relating a modelled reaction with a database entry (Step 3). These data-paths are then exhaustively searched for all measured kinetic rates of a reaction (Step 4)

The creation of large-scale metabolic models can be a time-consuming process and this is, in part, due to the manual collection of kinetic rates from online databases that use different naming conventions for reactions and models. To overcome this burden we have created a subroutine that is able to convert between naming structures of models and databases to exhaustively search for all possible measurements of kinetic rates from online databases (Fig. 2). In Additional file 1 we provide the pseudocode for this routine and discuss possible extensions to further constrain the database search for particular cases. The output of the script is a table that is required for input into the PB stage of the computational workflow [34, 41].

In Step 1 of Fig. 2, the databases a user wishes to search are registered. It is noted which kinetic rates they contain and how they can convert reaction or component IDs (identifiers, see below) to these kinetic rates or other IDs. The default databases used in this study are listed in Table 1. In Step 2 (Fig. 2), the SBML model is parsed such that names and identifiers of reactions or metabolites are obtained. This list of model components (both metabolites and reactions) plus the parameters we wish to find represents a set of *task* objects that are inputs into Algorithm 1 (Additional file 1). Based on these set of *tasks*, the online databases are searched exhaustively to find all experimentally-measured rates related to the metabolite reactions of interest.

**Table 1:** Overview of databases currently used in the pipeline

Database	Purpose	Reference
BRENDA	Michaelis constants, catalytic rates	[29]
SABIO-RK	Kinetic rates	[30]
eQuilibrator	Equilibrium constants	[31]
Rhea	Identifier translation	[42]
MetaCyc	Identifier translation	[43]
MetaNetX	Identifier translation	[44]

One issue with using online databases is that different sources use different naming conventions, e.g. BRENDA uses EC numbers, whilst eQuilibriator uses KEGG identifiers [25-27] to classify reactions. Consequently, methods of integrating these identifiers and translating from one database to another are required [29–31]. Thus in Step 3 (Fig. 2) a set of possible paths is constructed that links online data values to the rates needed to complete the model. The Rhea, MetaCyc and MetaNetX databases are used here to translate identifiers [42–44]. For example, assume we have a reaction name and we wish to obtain an equilibrium constant, *k*^*eq*^, then one possible path would be:

$$\begin{array}{*{20}l} &\mathrm{Name\ (Glucose-6-phosphate\ isomerase)}\\ &\qquad\qquad\qquad\qquad\rightarrow \mathrm{MetaCyc\ ID\ (ENZRXN-2863)}  \\ &\qquad\qquad\qquad\qquad\rightarrow \mathrm{Rhea\ ID\ (11816)}  \\ &\qquad\qquad\qquad\qquad\rightarrow \mathrm{KEGG\ reaction\ ID\ (R00771)}  \\ &\qquad\qquad\qquad\qquad\rightarrow \text{eQuilibrator}\ k^{eq} \mathrm{ (0.361)}.  \end{array} $$

Each of these pathways are exhaustively examined (Step 4, Fig. 2) until all the useful experimental measurements have been obtained. Full provenance and, if available in the original database, literature references are saved and can be manually examined if needed, for example, in the case of conflicts between identifiers.

#### Parameter balancing (step 5, Fig. 1)

Upon obtaining measured kinetic rates from online sources (and combining these with experimental measurements), a table of found values and their reference is automatically constructed, which is used as an input into the PB software. In order to improve the scalability of the parameter balancing algorithm and to support the parameter balancing of large genome scale models, the parameter balancing algorithm was implemented directly into the pipeline based on the original implementation by Lubitz et al. [34]. Here we shall briefly review the PB method and further details can be found in [33, 34, 41].

The principle of PB is to relate measured constants with unknown values via the Haldane relationships and Wegscheider conditions that ensure kinetic rates are thermodynamically feasible whilst reactions take place in an ideal solution [33]. These relationships are able to relate equilibrium constants, *k*^*eq*^, to catalytic rates, *k*^*cat*±^ (s^-1^), maximal velocities, *v*^*max*^ (mmol ·s^-1^), Michaelis constants, *k*^*M*^ (mM), enzyme concentrations *u* (mM), and standard chemical potentials, *μ*° (kJ ·mol^-1^) via

$$\begin{array}{*{20}l} \ln{k_{j}^{eq}} &= -\frac{1}{RT}\sum_{i}n_{ij}\mu_{i}^{\circ},  \\ h_{j}\ln{k_{j}^{eq}} &= \ln{k_{j}^{cat+}} - \ln{k_{j}^{cat-}} + \sum_{i}h_{j}n_{ij}\ln{k_{ji}^{M}},  \\ \ln{k_{j}^{cat\pm}} &= \ln{k_{j}^{V}} \mp \frac{h_{j}}{2}\sum_{i}n_{ij}\left(\mu_{i}^{\circ}/RT + \ln{k_{ji}^{M}}\right),  \\ \ln{v_{j}^{max\pm}} &= \ln{u_{j}} \,+\, \ln{k_{j}^{V}} \mp \frac{h_{j}}{2}\sum_{i}n_{ij}\left(\mu_{i}^{\circ}/RT \,+\, \ln{k_{ji}^{M}}\right),  \end{array} $$

where *k*^*cat*+^ is the forward catalytic rate, *k*^*cat*−^ is the backwards catalytic rate, $k_{j}^{V} = \sqrt {k_{j}^{cat+}k_{j}^{cat-}}$ (s^-1^) is the geometric mean of catalytic rates, *h*_*j*_ is the cooperativity factor for sigmoidal kinetics, *n*_*ij*_ is the stoichiometric coefficient of metabolite *i* in reaction *j*, *R* is the gas constant (J ·mol^-1^ ·K^-1^) and *T* is the temperature in Kelvin [34].

Furthermore, one can relate chemical potentials, *μ* (kJ ·mol^-1^), and reaction affinities, *R*^*A*^, to metabolite concentrations, *c* (mM), using

4 $$\begin{array}{*{20}l} \mu_{j} &= \mu_{j}^{\circ} + RT\ln{c_{j}}, \\ R^{A}_{j} &= -\Delta_{j}G = -\sum_{i}n_{ij}\mu_{i},  \end{array} $$

where *ΔG* is the chemical potential through a reaction [34]. The concentrations, *c*, obtained from the balancing routine are used as initial conditions when simulating the resulting ordinary differential equations (see below).

**Fig. 3:**
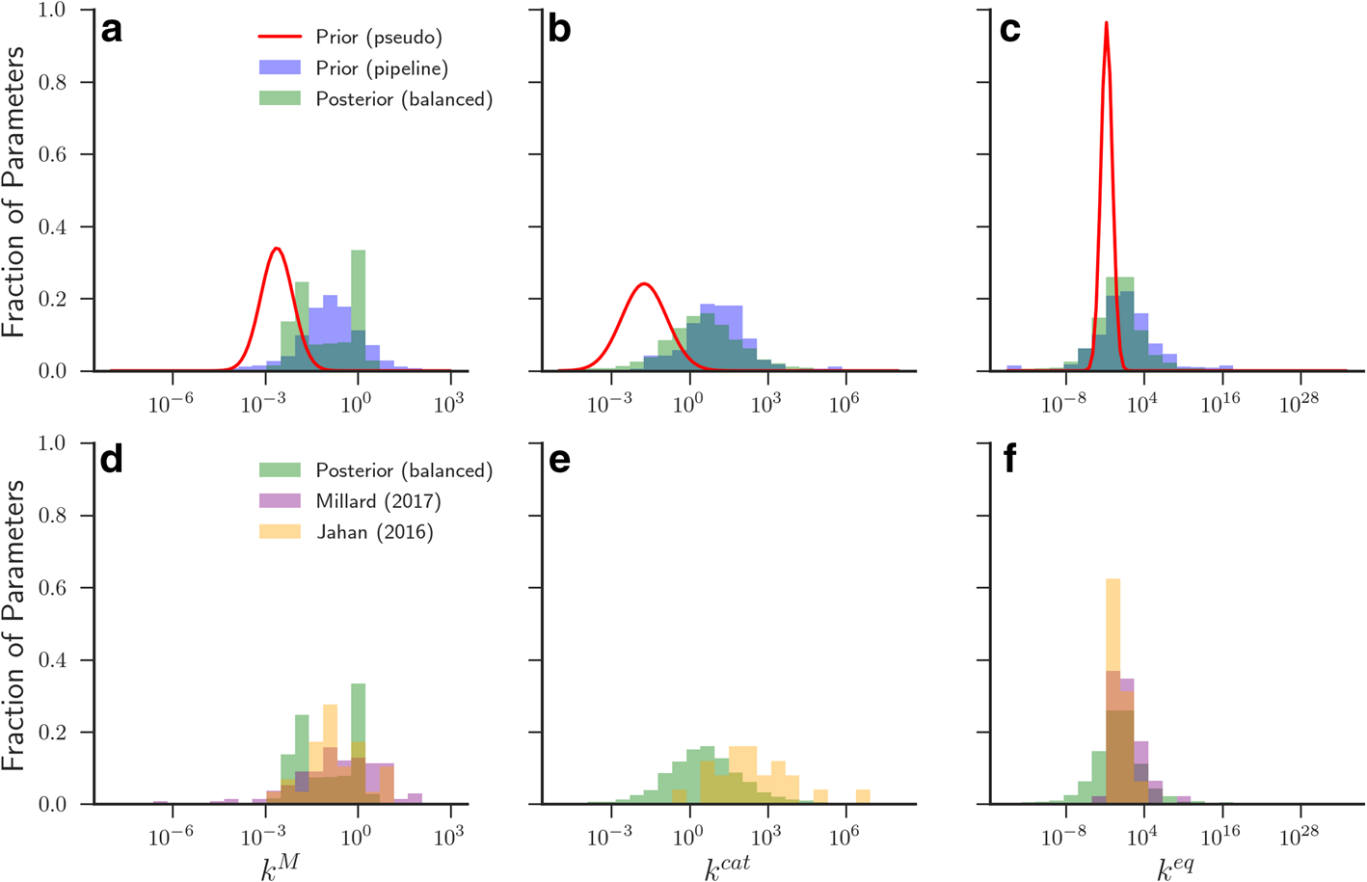
Obtained prior and posterior distributions for parameters in the *E. coli* iJO1366 genome scale model. Examples of distributions obtained for median **a** & **d** *k*^*M*^, **b** & **e** *k*^*cat*^ and **c** & **f** *k*^*eq*^ values in the *E. coli* iJO1366 genome scale model from the PB method and from parameter optimisation routines in published models. **a** — **c** The blue distributions are prior distributions obtained from databases, the red distributions are the pseudo values used when there is no prior information, and the green distributions are posterior distributions obtained after balancing. **d** — **f** The green distributions are the posterior distributions shown in (**a** — **c**). The yellow distributions are taken from [55]. The pink distributions are taken from [56]. Only parameters with prior information resulting from the parameter search are included in the comparison

Based on these relationships, any unmeasured values can be calculated directly given that a subset of the system parameters are known or can be estimated. This is the principle behind PB. By bringing together as much information as possible into prior distributions, Bayesian approaches are used to obtain the maximum likelihood estimates for kinetic rates that follow the thermodynamic relationships above. This results in posterior distributions for each kinetic rate in the metabolic system (e.g. Fig. 3, green distribution), providing a feasible range for these parameters [34]. However, in some cases where a system has not been well characterised, prior information for certain values may not be found within the databases or measured experimentally. In this case a pseudo-prior distribution is used to approximate the range of values one may expect to see experimentally (compare blue and red distributions in Fig. 3a-c as an example). Further details about the construction of our pseudo-prior distributions can be found in Additional file 1.

#### Mathematical translation (step 6, Fig. 1)

The next step of our framework is to translate the reaction network into a set of ordinary differential equations (ODEs). To do this we translate every reaction into a flux term, as in Eq. 3, using libSBML [40, 45] and Sympy [46]. For simplicity we use only the common modular rate law [33] based on the reversible Michaelis-Menten approximation

5 $$\begin{array}{*{20}l} &{}v\left(\{\mathbf{a},\mathbf{b},\mathbf{x}\},\mathbf{k}\right) = u_{l}\, f_{reg}\left(\mathbf{x},\mathbf{k}\right)\times\\ &{}\frac{k^{cat+}\prod_{i}\left(a_{i}/k_{a_{i}}^{M}\right)^{\alpha_{i}}-k^{cat-}\prod_{j}\left(b_{j}/k^{M}_{b_{j}}\right)^{\beta_{i}}}{\prod_{i}\left(1+a_{i}/k^{M}_{a_{i}}\right)^{\alpha_{i}}+\prod_{j}\left(1+b_{j}/k^{M}_{b_{j}}\right)^{\beta_{j}} -1 + D_{reg}(\mathbf{x},\mathbf{k})}, \end{array} $$

where

6 $$\begin{array}{*{20}l} f_{reg}(\mathbf{x},\mathbf{k}) &= \prod_{\{x_{i}:\mathbf{x_{A}}\subseteq\mathbf{x}\}}\frac{x_{i}/k^{M}_{i}}{1+\left(x_{i}/k^{M}_{i}\right)}\prod_{\{x_{j}:\mathbf{x_{I}}\subseteq\mathbf{x}\}}\frac{1}{1\,+\,(x_{j}/k^{M}_{j})},  \\ D_{reg}(\mathbf{x},\mathbf{k}) &= \sum_{\{x_{i}:\mathbf{x_{A}}\subseteq\mathbf{x}\}}\frac{k^{M}_{i}}{x_{i}} + \sum_{\{x_{j}:\mathbf{x_{I}}\subseteq\mathbf{x}\}}\frac{x_{j}}{k^{M}_{j}}, \end{array} $$

with *x*_*A*_ representing the vector of components that activate the reaction and *x*_*I*_ the vector of components that inhibit the reaction [33, 47]. Here, *f*_*reg*_ is known as allosteric reaction regulation, whilst *D*_*reg*_ is specific reaction regulation [33]. Thus, Eqs. 5 and (6) represent a generalised version of Eq. 3.

Consequently, the reaction network is translated into ODEs of the form

7 $$ \frac{d\mathbf{x}}{dt} = \mathbf{S}\cdot\mathbf{v}(\mathbf{x},\mathbf{k}) = v_{f}(\mathbf{x},\mathbf{k}) - v_{r}(\mathbf{x},\mathbf{k}),  $$

where *v*_*f*_ and *v*_*r*_ are the sum of forward and reverse fluxes, respectively. In the case of irreversible reactions, their forward or reverse rate can be fixed to zero to remove these fluxes. To include the effect of compartmentalisation one needs to appropriately rescale concentrations within the different subsystems. In the pipeline this can be done by assigning the metabolite to a compartment directly in the initial SBML file, which will be preserved in the final output model and can subsequently be compensated for by the numerical integration tool of choice.

#### Simulation (step 7, Fig. 1)

Now that we have a set of ordinary differential equations and kinetic rates to determine their functions, we can simulate the metabolic models given a set of initial conditions. Furthermore, system inputs can either be fixed constant or perturbed during simulation (e.g. a pulse of glucose uptake). Simulations were conducted using libRoadRunner [48] and Scipy [49]. As has been discussed previously, due to the size and stiffness of large genome-scale ordinary differential equation models, numerical instabilities can make simulation difficult as processes occur on different time-scales [5]. In all the presented results, we simulate the test models for 400s, providing a pulse in the level of one of the metabolites after 200s (Glyceraldehyde-3-Phosphate for the *L. lactis* and *E. coli* models). The initial conditions for the simulations are obtained from the PB routine, using the *c*’s in Eq. 4. The parameter values used are the median values obtained from each individual parameter distribution.

#### Model reduction (optional step)

The optional model reduction step is currently employed before Step 1 of our pipeline. To do this, we used the NetworkReducer algorithm that has been implemented in the MATLAB toolbox CellNetAnalyzer [35, 39]. NetworkReducer decreases the size of metabolic networks by iteratively removing the reaction with the lowest flux variability determined using Flux Variability Analysis (FVA) [24]. The iterative process ends when no more reactions can be removed without violating the behaviours of the full model (i.e. specific growth or production rates on a predefined medium). Finally, a compression step is used that compresses linear pathways into a single effective reaction. We applied both the pruning measure that removes reactions with the smallest range of possible fluxes whilst maintaining phenotypes and protected pathways, and the network compression step that lumps reactions from connected pathways to the subsystem of interest into a single reaction. For details on how the algorithm achieves this, please see [35]. In the analysed networks, we set parts of the glycolysis pathway to be protected, whilst a resulting growth rate within 1% of the growth rate obtained from the full model had to be maintained. The growth rate of the full system was approximated using COBRApy [50].

### Test models

To highlight the utility of our pipeline, we will illustrate our analysis for multiple systems. In order to fully analyse the accuracy of the pipeline, we have used the central metabolic pathway of *L. lactis* [1]. The initial mathematical model of this system is already in the correct form of Eq. 7 for most of the reactions and provides ranges for kinetic rates within the system that we can include in our pipeline. To show that the pipeline can also be used for larger scale systems, we use the *E. coli* core and the iJO1366 genome scale models [51, 52] in combination with the optional reduction steps.

In order to show how our automated search for kinetic rates improves the number of parameters found from online databases, we also included the *S. cerevisiae* model iTO977 and the iJO1366 *E. coli* model that is well annotated relative to other species [52, 53].

Finally we have included randomly generated reaction networks in order to facilitate testing effects of including regulation and compartmentalization. See Additional file 1 for details on the generation of these models.

### Pipeline analysis tests

**Fig. 4:**
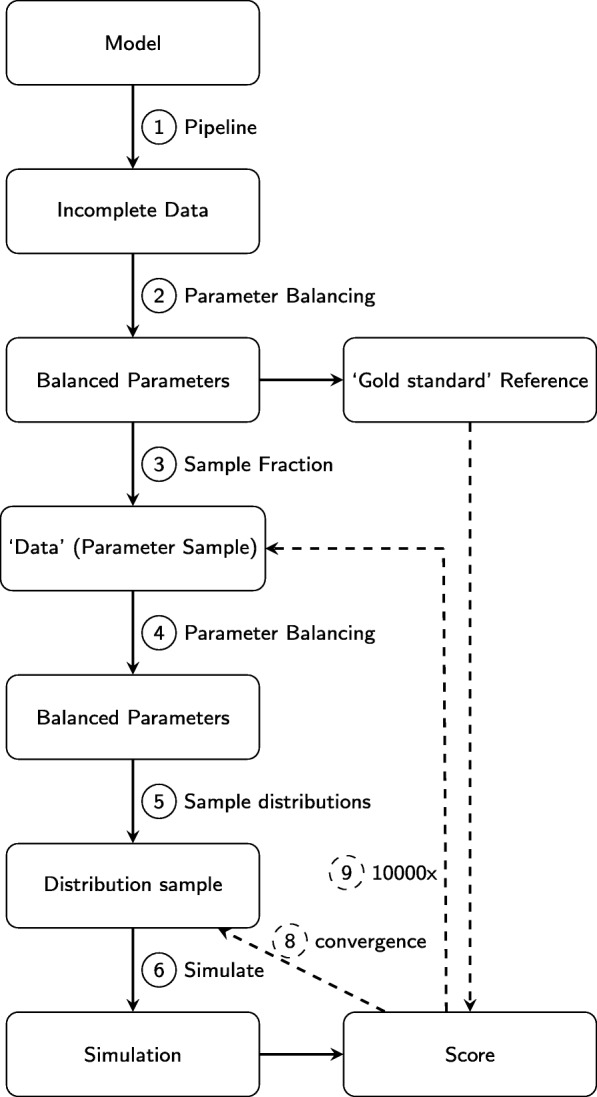
Analysing the robustness of DMPy. Schematic explanation of tests performed to assess the robustness of our pipeline. Using prior information for kinetic rates in the system (1), a model is created whereby the distribution of all parameters is known (2). The median of all parameter distributions is then used to simulate a ‘gold standard’ set of time-series of system components. We then use a subset of the known parameter distributions (3) as an input to create a new model of metabolism (4), whose parameter distributions are sampled (5) and the resulting dynamic model is simulated (6) and compared to the ‘gold standard’ reference. When the score converges (8), it is saved and a new fraction of the ‘Data’ parameters is drawn (9)

In order to test the accuracy and reproducibility of our framework we generated a simulated dataset from a parameter balanced model of our tested reaction networks. To obtain the model we input the reaction network into our pipeline such that we have an idealized ‘gold standard’ *in silico* system with fixed values (using the medians of the balanced distributions) for every parameter in the network (Fig. 4). Using randomized subsets of the fixed parameter values as prior inputs into a second round of PB we simulate the availability of a subset of the parameter data. The subset of data is used to create a new model through parameter balancing and is subsequently simulated. Finally, the mean square error is calculated compared to the simulation of the ‘gold standard’ reference model. We then performed the following tests:

- how many times the posterior distributions need to be sampled before there is convergence in the mean square error between the samples and the simulated ‘gold standard’ data;
- the amount of prior distributions that need to be known to obtain minimal differences between the model obtained from the pipeline and simulated data, and;
- the effect of altering the width of the posterior distribution sampled from.

Convergence was tested by taking the standard error of deviation relative to the mean score. After an initial period of 25 samples, the simulation was marked as converged when $(\sigma _{s} / \sqrt {n}) / \mu _{s} < 0.05$, where *σ*_*s*_ is the standard deviation of the score, *μ*_*s*_ is the mean score and *n* is the number of simulations. If convergence was not obtained within 10000 samples, the simulation run was halted and marked as unconverged.

## Results

### Parameter gathering is improved by including identification translation and previously estimated rates

The first step of the computational pipeline (Fig. 1) is to automatically obtain distributions for kinetic rates from online databases. We tested the automated search using four models - the *E. coli* core and iJO1366 models, *S. cerevisiae* model iTO977 and the central metabolic pathway of *L. lactis* [1, 51–53]. Furthermore, in order to compare the influence of automatically translating naming conventions, we present the results with and without the usage of Rhea, MetaCyc and MetaNetX databases [42–44]. The total number of parameter values found from each database are shown in Table 2 with the list of values provided in Additional file 2.

**Table 2:** Coverage of kinetic rates obtained from the databases with (+) and without (-) the use of identifier translators

Model	Database	*k* ^*M*^		*k* ^*cat*^		*k* ^*eq*^	
		+	-	+	-	+	-
*E. coli*	# rates	10183		2583		2583	
iJO1366	BRENDA	637	591	286	259	/	/
	eQuilibrator	/	/	/	/	491	0
	SABIO-RK	228	0	4	0	0	0
	# found	700	591	290	259	491	0
*E. coli*	# rates	380		95		95	
Core model	BRENDA	29	0	10	0	/	/
	eQuilibrator	/	/	/	/	37	0
	SABIO-RK	10	0	2	0	0	0
	# found	33	0	12	0	37	0
*S. cerevisiae*	# rates	5509		1612		1612	
iTO977	BRENDA	342	330	113	108	/	/
	eQuilibrator	/	/	/	/	450	0
	SABIO-RK	104	96	3	0	0	0
	# found	370	356	116	108	450	0
*L. lactis*	# rates	73		21		21	
	BRENDA	2	2	1	1	/	/
	eQuilibrator	/	/	/	/	12	0
	SABIO-RK	2	2	0	0	0	0
	# found	3	3	1	1	12	0

Rhea, MetaCyc and MetaNetX databases were used for identifier translation

If multiple values were found for the same reaction rate then this was only counted once

If the rate is not available from this database, it is noted with /

From Table 2, there are two results that should be highlighted. First, upon inclusion of the identification translating databases, the number of parameters for which values are found always increases, suggesting that our automated searching strategy functions correctly and finds a larger subset of parameter values within the databases. Second, as a percentage of the total number of kinetic rates being searched for, there is a higher fraction of equilibrium constants, *k*^*eq*^, available online than other reaction parameters. Unlike enzyme specific properties such as the catalytic rate constant or the Michaelis constant, the equilibrium constant is only specific to the chemical reaction and the environment in which it takes place. Thus they are more readily available then other kinetic rates, which have to be individually measured for each enzyme. Furthermore, the equilibrium constants can be predicted using methods such as component contribution [54], as implemented by eQuilibrator [31], which can also take into account factors such as the pH and ionic strength of the cellular environment. Despite this minor success, though, the number of measured reaction rates is only a small fraction (roughly 1%) of the total needed to fully parameterise a model. We discuss in Additional file 1 how our parameter searching algorithm can also filter results for specific experimental conditions, such as pH or temperature. This suggests that efforts to experimentally determine these rates in a high-throughput manner should be continued in order to improve our knowledge of parameter distributions required for model development. However, for all unmeasured kinetic rates, the PB algorithm constructs pseudo-distributions to estimate the feasible range of certain parameters under specified experimental conditions. Alternatively, parameter estimation methods can be used when the appropriate data is available (see Discussion).

In Fig. 3a-c, we show the distribution of each individual parameters median value obtained from the parameter distributions before and after using the PB algorithm. When looking at the distribution of median values found for the iJO1366 *E. coli* model, we observe that the distributions both before and after balancing are approximately log-normally distributed (Fig. 3a-c), as is assumed by the PB algorithm. It is notable that the resulting posterior distribution of median values shows good overlap with parameter values used in models of *E. coli* central carbon metabolism that have been obtained using different parameter optimisation methods (Fig. 3d-f) [55, 56].

### The amount and quality of prior knowledge influences accuracy of resulting model

Given the observation that altering the amount and quality of experimentally measured or computationally estimated parameter values influences the prior and posterior distributions obtained we wished to understand the effects of these changes on system dynamics more thoroughly. In order to do this we took the parameter balanced model of *L. lactis* metabolism and generated time-series data of all compounds. This simulated ‘gold standard’ reference dataset was then used to compare the generated dynamics obtained using different varieties of input into our framework (Additional file 1: Figure S1). Notably, the dynamics are qualitatively different with our pipeline compared to the original model (compare blue line with orange and green lines) [1]. In our model, a glucose pulse leads to a decrease in FBP and G3P concentrations. This reflects two factors. First, the mathematical form of reactions in our model is constrained leading to a different model structure to that published by [1]. Second, in our balanced model we find Michaelis-Menten constants and maximal velocity rates that differ from the original model. The net effect of these alterations is that the balance between F6P and FBP conversion is altered such that, after a glucose input, our model simulates an increase in FBP to F6P conversion rather than FBP to G3P. This leads to a drop in FBP and a corresponding decrease in G3P concentrations.

**Fig. 5:**
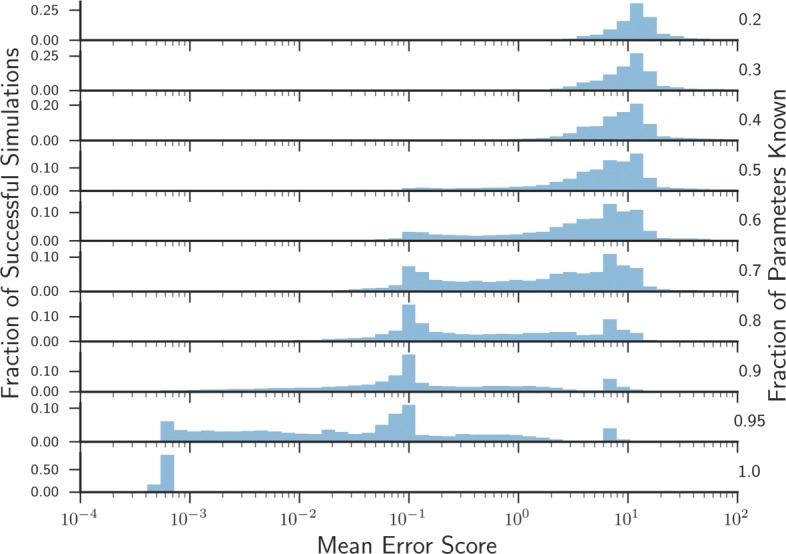
Analysing the robustness of DMPy. Using prior information for kinetic rates in the *L. lactis* system, a model is created whereby the distribution of all parameters is known. From these simulations we select a subset of the data to act as a ‘gold standard’ set of experimentally measured time-series of system components. We then use a subset of the known parameter distributions as an input to create a new model of *L. lactis* metabolism, whose dynamics are compared against the ‘gold standard’ dataset. Given a percentage of known parameter values used as input into the pipeline, the posterior distribution is resampled until the mean error score between model simulations and the ‘gold standard’ dataset converges (Fig. 4). The mean error score is recorded for this subset of parameters, after which a new subset is chosen and the process is repeated (n=10000). Only successfully converged subsets are shown. See Additional file 1: Figure S2 for more information

**Fig. 6:**
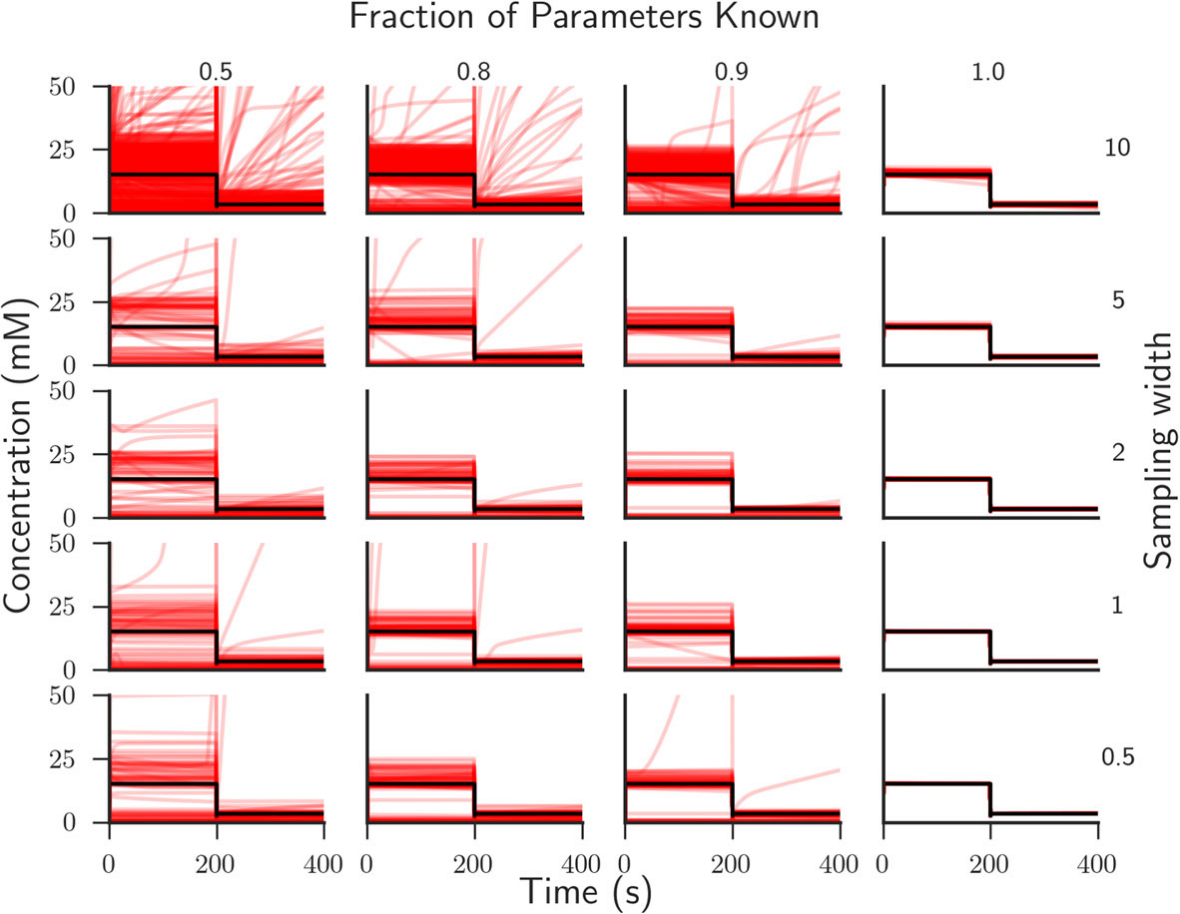
Simulated dynamics are increasingly variable when using low quality data. The effect of altering the sampling width of the posterior distributions on output simulations is observed given differently sized subsets of known values of the kinetic rates. Models were simulated for 400 s with a G3P pulse after 200 s. The distributions were sampled 100 times (red) and overlaid on the reference ‘gold standard’ simulation (black)

We used the ‘gold standard’ data to perform three tests of the computational pipeline. First, we analysed the average error between the simulated dataset and system dynamics from the output model obtained from the pipeline given only a certain amount of kinetic rates are known (Fig. 5). Second, we determined how many times the posterior distributions need to be sampled before the average difference between output simulations and the ‘gold standard’ data converges given different amounts of prior information (Additional file 1: Figure S2). Finally, we looked at the effect of altering the sampling width of the posterior parameter distribution on pipeline output (Fig. 6).

As one would intuitively expect, our analysis shows that having a larger number of experimentally measured or optimised kinetic rates results in system dynamics that better reflect the underlying biological network (Fig. 5). However, it is interesting to note that the number of samples required before convergence of the mean square error of the system dynamics compared to the ‘gold standard’ increased with the number of known parameters (Additional file 1: Figure S2, green lines). This likely reflects that, when little information is known, most sampled parameter sets from the posterior distribution yield equally poor simulations. For random networks we obtained more intuitive results where more accurate posterior distributions require fewer samples to converge (Additional file 1: Figure S2, orange lines).

Finally, it can be observed that there are multiple distinct peaks in the error distributions (Fig. 5). This can be an indication of local minima in the parameter landscape where the structure of the network has an inherent tendency towards certain concentration states. In Additional file 1 (Figs. 2 and 3) the same analysis was performed on a randomly generated metabolic network. This supports the observation that with less than approximately 80% of the parameters known the simulation error steeply increases.

In addition, it has to be noted that not only decreasing the fraction of known parameters causes an increase in error, but also increasing the sampling width of the posterior distribution (Fig. 6). This indicates that having high quality measurements or estimates of the parameters is another essential factor. However, simulated dynamics are more sensitive to decreased fractions of known parameters than a decrease in parameter quality (compare the improvement of simulation accuracy across rows of Fig. 6 to down columns). Thus, having a rough estimate of most parameters can be considered better than knowing few parameters with high accuracy.

### Performing flux-based model reduction techniques alters system dynamics

One aspect of model construction that may ease the requirement for measuring/approximating a large number of parameter sets is to reduce the size of metabolic networks being analysed. Thus, if the size of the metabolic network can be reduced, whilst maintaining an accurate description of experimentally observed phenomena, then the resulting mathematical model will contain lower numbers of reactions and kinetic rates. We explored the effect of reducing model size such that systems still maintained an optimal flux through the system using NetworkReducer (see Implementation and methods) [35]. This method allows one to not only prune reactions from the metabolic network that do not influence the optimal flux vector, but also to compress side-reactions into single, lumped reaction nodes.

**Fig. 7:**
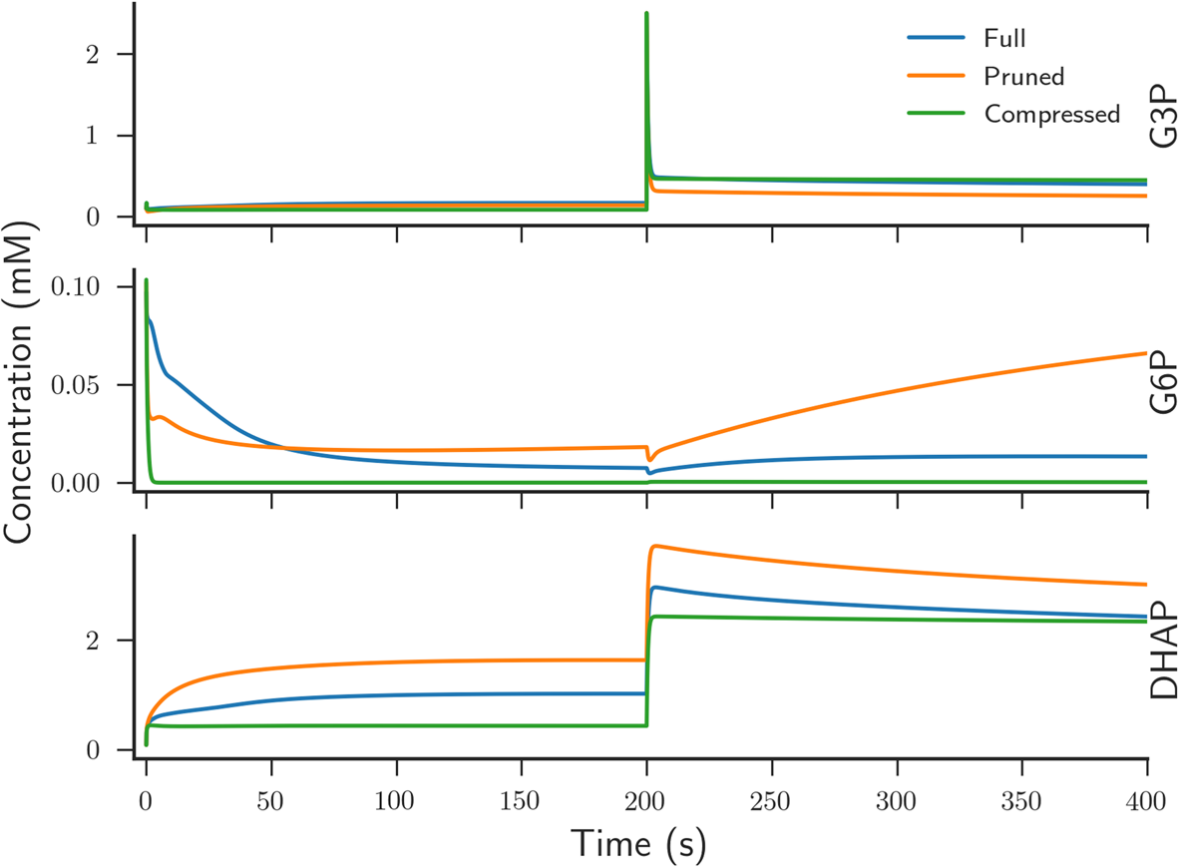
Flux-based model reduction alters system dynamics of metabolic components. Simulated dynamics of *E. coli* metabolism obtained in the absence of model reduction of the *E. coli* core genomic model (blue line), with a pruned model (red line) and with a pruned and compressed system (green line). Time-series for G3P, G6P and DHAP are shown. Models were simulated for 400 s with a G3P pulse after 200 s

We applied both methods to the *E. coli* core metabolic model [51] and used our pipeline to generate dynamic models for the complete and the two reduced models (Fig. 7), using the median of the balanced parameter distributions for the parameter values. Note that lumped reactions do not correspond to physical reactions any longer and, as a result, our pipeline will not find any parameters for these reactions. The pruned model, where reactions having a low variability flux are iteratively removed, contained 38 reactions and 40 metabolites compared to the original models 95 reactions and 92 metabolites. The compressed model, where in addition to the pruning step linear pathways are compressed into a single reaction, contained 18 reactions and 19 metabolites.

Figure 7 highlights that system dynamics can be quantitatively altered through the use of NetworkReducer. Furthermore, there are clear qualitative differences between the dynamics of the full and reduced models for several system components. Therefore, this suggests that using flux-based constraints for model reduction may not be the best method when one is interested in system dynamics and that networks should be reduced by other means (see Discussion).

### Comparing pipeline utility for models of varying size

To highlight the utility of our complete pipeline, we compare the time and memory required to obtain a dynamic model of various metabolic models (Table 3). To prevent bias in our comparisons, all models were created once using offline libraries obtained from the online databases. Thus, any time delays due to accessing the online databases does not influence our results. The models range from small, core parts of metabolism (the *E. coli* core model and the model of *L. lactis* metabolism) and larger, genome-scale metabolic models (for *E. coli* and *S. cerevisiae*) [1, 51–53]. These systems range from 10’s of reactions and metabolites to 1000’s and, thus, provide a good range of examples for which our pipeline is designed to be used for. Without including the optional model reduction step, we found that we could obtain a system of ordinary differential equations with balanced parameters for the largest systems within 1 hr, whilst it takes a matter of minutes to obtain smaller models. Notably, the majority of time and computer power required to create a model stems from searching for and balancing the kinetic rates (that requires mathematical transformations of large matrices [34]) from the searched databases. Compared to manual curation of parameters and developing a large system of differential equations, we believe this is a vast improvement in speed to obtain an initial dynamic model of metabolism.

**Table 3:** Comparison of run times and memory usage of pipeline for models of different sizes

Model	Reactions	Peak	Parameter	Parameter	Model	Total
	(metabolites)	memory	search	balancing	building	
		(GB)	(min:sec)	(min:sec)	(min:sec)	(min:sec)
*E. coli*	2583	29.6	22:05	33:14	2:12	57:33
iJO1366	(1805)					
*E. coli*	95	0.2	0:58	0:04	0:07	1:10
Core model	(92)					
*S. cerevisiae*	1612	19.1	13:18	11:44	1:14	26:18
iTO977	(2245)					
*L. lactis*	21	0.2	0:34	0:01	0:01	0:41
	(26)					

All values were calculated using 12 x 2.1 GHz cores of an AMD Opteron Processor 6272

Peak memory = maximum amount of RAM used

Databases were searched locally offline so that online connection speeds did not influence results

## Discussion

In this work we have introduced an automated computational pipeline that translates a genome-scale network of cellular metabolism into a parameterised set of ordinary differential equations that can simulate the dynamic behaviour of system components. Whilst this pipeline is inspired by the works of [5, 11], these steps have previously required manual efforts to collect appropriate datasets, reaction rates and to translate the metabolic network into mathematical equations. Thus, bringing these processes together into a single package, DMPy, will have beneficial consequences for many researchers interested in the dynamics of metabolic pathways.

The pipeline has three key steps and one optional (model reduction) process (Fig. 1). Upon parsing a genome-scale metabolic network, we have developed an algorithm that automatically searches through a set of prescribed online databases to find all available experimentally measured reaction rates (Fig. 2, Tables 1 and 2 and Additional files 1 and 2). Where parameters are unmeasured, pseudo distributions are constructed estimating the approximate value that these rates would take upon measurement. These parameter distributions based on our prior expectations are then used as an input into the previously published PB algorithm (Fig. 3) [34]. The resulting posterior parameter distributions then contain reaction rates that satisfy a range of thermodynamic criteria such as Haldane and Wegscheider relationships. Conveniently, the distribution of median parameter values that we use for model simulations closely resemble parameter distributions found using other methods to model *E. coli* carbon metabolism [55, 56]. The metabolic network is subsequently translated into a generalised set of differential equations using the reversible Michaelis-Menten approximation (Eqs. 5 and (6)) that can be simulated to explore the dynamics of metabolism [9]. We show the utility of our framework by analysing the results of the central metabolic pathway of *L. lactis*, randomized networks, and both the full and the core model of *E. coli* metabolism [1, 51, 52]. In the following subsections we shall describe where we think efforts could be made to improve the effectiveness of our initial pipeline.

### Precise measurements of kinetic rates in high-throughput shall improve model accuracy

We analysed the results of the algorithm in comparison to simulated ‘gold standard’ data for the central metabolic pathway of *L. lactis* that has been previously modelled using differential equations (Fig. 5) [1]. Notably, we found that the accuracy of parameterised dynamic models using our pipeline is dependent on the amount and quality of measurements of the kinetic rates within the *L. lactis* system. By resampling the posterior distributions 10000 times and computing the average error in comparison to the simulated dataset, we found that accurate estimates for >80% of the reaction rates are required to obtain an approximately close match between simulated and target metabolic time-series data (Fig. 5). Whilst we note that this result may differ for other systems, this finding is notable as it is often the case that less than 1% of the required reaction rates have been measured (Table 2). This suggests that efforts of obtaining measurements or estimates of reaction rates are necessary to help construct accurate parameterised dynamic models of metabolism.

One way of improving dynamic models is through improved high throughput measurement techniques. By altering the width of prior parameter distributions we can analyse the effect of measurement precision of kinetic rates whereby narrower prior distributions imply more precise measurements. What we observed was that increasing the sampling width of the prior distributions quickly led to erroneous dynamics being simulated from the resulting model and parameter set (Fig. 6). This was supported both when a high or low fraction of the required reaction rates had been measured. Since in vitro parameter measurements can often have errors of an order of magnitude or more compared to in vivo, great care has to be taken when directly integrating these measurements. Techniques, such as that by [32], aim to alleviate this problem by integrating multiple sources of data from different conditions and could be integrated into our pipeline. In conclusion, the experimental set-up used to obtain kinetic rates requires high precision in order to decrease the width of parameter distributions and improve model accuracy. We suggest that such an idealised high-throughput precise measurement method should be one of the key targets for future research in this area.

A second option to obtain good estimates of reaction rates from limited data is the use of parameter optimisation methods (as in [55, 56]). Recently, Fröhlich et al. have proposed a method that can find estimates for 100’s of parameters faster than previous methods [57]. Thus, one could include this method in our pipeline after the construction of the ODEs to fine-tune model dynamics. However, two aspects should be noted. First, any parameter estimation method should include constraints such that parameters satisfy the Haldane and Wegscheider relationships used in the PB algorithm (see Implementation and methods). Second, it is hard to predict how including such measures will increase the run time of our pipeline as presented in Table 3. Hence, finding an efficient parameter optimisation method for large systems is a key aspect of future research.

### Model reduction should incorporate knowledge of system dynamics

In this work we have shown how flux-constrained model reduction methods result in smaller systems that have qualitatively different dynamics to the original complete model (Fig. 7) [35, 36]. Essentially, these model reduction techniques look to remove reactions from larger networks that have little influence on the resulting reaction fluxes important for observed phenotypes. For example, in NetworkReducer (see Implementation and methods) [35], flux variability analysis (FVA) is used to find reactions that have minimal impact on resulting flux estimates. Thus, these minimal-impact reactions can be removed without altering system fluxes. However, since these methods are performed using the genome-scale reaction network, the model reduction step is performed before the creation of a dynamic model and, subsequently, does not take into account any information about temporal system dynamics. Consequently, we have found that the dynamics of metabolic components are influenced by flux-based model reduction techniques (Fig. 7). This suggests that any flux-based reduction method is only useful as a first approximation when no data is available. The reduced model could then be used to aid direct experiments to obtain better quality data for key components in the system — conveniently also aiding the parameter estimation problem discussed above.

This raises the issue of whether methods can be automated that are able to reduce a large-scale metabolic network to maintain specific dynamic requirements, such as a match to time-series data. One could envisage two possible techniques; first, by reducing the differential equations based on time-scale arguments (for example, the quasi-steady state assumption) [37], and second, by using flux-based reduction methods and appropriately re-approximating the parameters of reduced modules such that the remaining parts of the system are dynamically consistent with the full model. In the second instance this would require generating both a model of the full and reduced networks and then dynamically comparing their output. Thus, rather than developing a single model with our pipeline, two would need to be produced, thus increasing the computational time to generate a model. This is undesirable and, consequently, reducing dynamic models by focussing on a specific time-scale may be more appropriate.

Within biological systems, processes occur across a range of time-scales (roughly from femtoseconds to hours). Thus, one is generally interested in understanding what happens at a specific time-scale and ignoring or simplifying those processes that happen too quickly or slowly, consequently reducing the systems complexity [37]. Importantly, the speed of reactions could be approximated by their maximal velocity. Upon adaptation of our pipeline, one could in principle search for all component dynamics that occur either too quickly or slowly compared to the components of interest and fix their concentrations as constants within the system. This reduces the number of components and equations that require parameterisation and simulation. Such ideas are the focus of future work and pipeline developments.

### Expanding the mathematical functions within the model

One other limitation of our current pipeline is that every reaction is mathematically described by the same reversible Michaelis-Menten approximation, including transport reactions or genetic interactions. In Additional file 1, we have presented comparative effects of including compartmentalisation within metabolic networks and the effects of including or altering regulatory mechanisms (Additional file 1: Figures S4 and S5), automating a process of selecting mathematical functions from a library of specific reactions may also increase the accuracy of the resulting models. This would allow for the appropriate depiction of known transport reactions between compartments and the addition of transport regulators or saturation effects. Tools such as SBMLSqueezer [58], allow for automated selection of rate laws based on the components and annotation of the reaction and could be integrated into the pipeline at the model generation step, although it has to be considered how different rate laws can fit into the assumptions of the PB framework. Additionally, recent work by [59] shows how to generate a genome scale map of regulatory interactions of small molecules. This could further improve the quality of the generated networks, especially when starting from existing genome scale metabolic networks, where this information is often lacking.

Furthermore, by allowing a user to easily manipulate and alter mathematical functions, sub-modules of larger networks can be easily replaced by known, more-detailed kinetic models of specific pathways. Importantly, our pipeline allows users to fix parameter values by using narrow prior distributions. Consequently, the resulting models will maintain the dynamics of a more detailed model whilst allowing one to observe the effects of the metabolic pathway on a genome-scale reaction network. Future developments of this computational framework will aim to incorporate these ideas to provide more flexibility to users such that finer details can be added to large-scale models of metabolism.

## Conclusions

In conclusion, we have developed a modular framework that provides an initial approximation of temporal metabolic changes within a cell, which can easily be extended with additional data as required. Due to the modular approach, individual methods within the framework can be replaced or updated as they are enhanced. Furthermore, by generating more detailed data, we have shown that the accuracy of these dynamic models will improve given the current methods used within the pipeline. In addition to this, through the use of model reduction techniques and compartmentalisation within a cell, individual subnetworks or compartments within the dynamic model can be easily manipulated and replaced as metabolic pathways are studied in more detail. We envision that this framework will be of great use to the metabolic community as attempts continue to unravel the complex relationship between system inputs and physiological outputs that are relevant for the bio-industry sector.

## Availability and requirements

DMPy can be found at gitlab.com/wurssb/DMPy along with all computer scripts and models used in this study. DMPy is written in Python, further implementation details can be found in Additional file 1.

**Progject name:** DMPy.

**Homepage:** gitlab.com/wurssb/DMPy.

**Programming Language:** Python.

## Additional files

Additional file 1 Appendix. Implementation details of computational workflow; pseudo-code for automated parameter search subroutine; methods of constructing prior distributions of kinetic rates; compartmentalisation and regulation; method of generating random reaction networks; Supplementary Figures. (PDF 827 kb)

Additional file 2 Parameter values. Reaction rates that were obtained by our automated database search for different metabolic systems. (XLSX 510 kb)
