# Supplementary material for: DMPy: a Python package for automated mathematical model construction of large-scale metabolic systems
Source: BMC Syst Biol. 2018 Jun 19;12:72. doi: 10.1186/s12918-018-0584-8 (PMC6006996; doi:10.1186/s12918-018-0584-8)
Supplement: Supplementary file 1 — Appendix. Implementation details of computational workflow; pseudo-code for automated parameter search subroutine; methods of constructing prior distributions of kinetic rates; compartmentalisation and regulation; method of generating random reaction networks; Supplementary Figures. (PDF 827 kb) [file 12918_2018_584_MOESM1_ESM.pdf]

DMPy: Additional File

Robert W. Smith[*†], Rik P. van Rosmalen[*], Vitor A. P. Martins dos Santos[*†], Christian Fleck[*‡]

Contents

1 Implementation and inputs for *DMPy* **2**
1.1 Software requirements 2

2 Parameter search **3**

3 Constructing pseudo distributions **5**

4 Compartmentalisation and regulation **6**

5 Generating Random Metabolic Networks **7**

5 References **10**

6 Supplementary Figures **11**

[*]Laboratory of Systems & Synthetic Biology, Wageningen UR, PO Box 8033, 6700 EJ Wageningen, The Netherlands.
[†]LifeGlimmer GmbH, 12163 Berlin, Germany
[‡]Corresponding author: christian.fleck@wur.nl.

1 Implementation and inputs for *DMPy*

DMPy is available from the Wageningen UR gitlab at `gitlab.com/wurssb/DMPy`. Full installation instructions and examples can be found in the repository README file.

Required input is the annotated stoichiometric model in SBML format and the name of the organism. Existing experimental values for parameters can also be provided, as is shown in one of the examples. Note that the use of the BRENDA database requires a (free academic) account, the username and password will be prompted for when required. Examples can be found in the file `tests.py`.

1.1 Software requirements

The easiest way to get all Python modules up and running is using the Anaconda Python distribution (version 2.7.x) which comes with precompiled packages for Windows, OS X or Linux. You can find it at `https://www.continuum.io`. Otherwise, modules can be installable with pip, the Python package manager, or through the methods described by the documentation of the module in question.

Use either conda or pip to install the following modules. Install with: `pip install $module$` or `conda install $module$`.

- `numpy`
- `scipy`
- `libroadrunner` (for simulating examples, when using conda use `roadrunner` from channel `-c sys-bio`)
- `sympy` (For creating the kinetic formulas)
- `matplotlib` (For plotting results)
- `soappy` (For accessing Brenda)
- `requests` (For accessing Sabio-RK)
- `libsbml` (For reading/writing sbml, when using conda the package name is `python-libsbml` from channel `-c SBMLTeam`)
- `cython` (For speeding up part of the balancing procedure, requires a C compiler)
- `NetworkX` (For generating random networks)
- `pint` (For converting units)

2 Parameter search

The parameter search step in the pipeline is implemented to aid model construction by automatically searching for parameters based on the content of the SBML model file. For each reaction and metabolite, the notes and annotation sections of the SBML entry are searched for identifiers. Currently this is limited to *Chebi*, *Inchi*, *Kegg Compound* and *Pubchem substance* identifiers for metabolites and *Kegg Reaction*, *Reactome*, *Rhea* and *Sabio-RK Reaction* identifiers for reactions, as these were commonly found in the models used for testing the pipeline. However, different identifier patterns can be implemented if required by altering the *transforms* input into Algorithm 1. These identifiers, together with the names of the reactions and metabolites, are then used to create task objects which are used as input for the parameter search as described in Algorithm 1.

The *transforms* inputs, implemented as Python objects, takes a set of identifiers required for certain databases and the parameters that need to be searched for, and a Python function that retrieves parameter values from online databases using the listed identifiers. The *task* inputs, also implemented as Python objects, contain the reactions and/or metabolites we are interested in (e.g. determined by reaction names or metabolite Kegg identifiers) and which parameters we wish to find (e.g. Michaelis or equilibrium constants). The combination of *tasks* and *transforms* can then be viewed as a graph where the *transforms* form paths between *task* objects that need to be exhaustively searched to find all possible parameter values (Algorithm 1).

After the search for parameter values, all found parameter values are filtered for unit mismatches or if they are obtained from other strains (e.g. knock-out or knock-in strains). Furthermore, duplicate entries are removed, if they have both the same value and result from the same source publication, as marked by the PubMed Identifier or other source identifiers as noted in the database of origin. One can, finally, also extend our algorithm to filter the resulting parameter sets to only include those values obtained under specific experimental conditions (e.g. pH or temperature). Additionally, both the PB method and values obtained from eQuilibrator can also take into account these environmental factors [1–3].

Due to the flexibility of the approach, the parameter search could be expanded in the future to account for different parameters, identifiers or databases as highlighted above. Other ways to retrieve more parameters, especially for non-model species, could be to include results from related species. Thus, reactions from multiple species are searched for at the same time. The rates that are then obtained from increasingly distally-related species are penalised using an error penalty depending on the evolutionary distance between organisms. In order to achieve this, one would need to incorporate a pre-step to our search algorithm to find extra reactions

from other species (that could be included within the *transform* input) and a post-step to penalise results obtained from evolutionary-distant related species. Another issue is when little information is known about a particular reaction. A user could alter our algorithm to find extra information by searching for sets of products and substrates, or through the sequence or structural similarity of catalysing enzymes. Finally, another interesting addition would be to automatically retrieve candidates for reaction inhibition or activation, as these are often ignored in the genome scale metabolic model but can be important for accurately describing the system dynamics.

Algorithm 1 Pseudo code for exhaustive search of parameter values

```
function ParameterSearch(tasks, transforms)
    # Create exhaustive search path
    for task ← tasks do
        possible ← ∅
        repeat
            for transform ← transforms do
                if requirements_transform ⊆ (task_identifiers ∪ possible) then
                    possible ← possible ∪ outputs_transform
                    Append transform to path
        until No new transform added

        # Prune unnecessary transforms from the search path
        repeat
            used ← {identifier ∈ transform_requirements for transform in path}
            used ← used ∪ task_wanted
            for transform ← paths do
                if transform_outputs ∩ used = ∅ then
                    Remove transform from path
        until No new transform removed

        # Follow path and find identifiers and parameter values
        for transform ← paths do
            if requirements_transform ⊆ task_identifiers then
                result ← transform(task_identifiers)
                if result then
                    task_identifiers ← task_identifiers + result_identifiers
                    task_parameters ← task_parameters + result_parameters
    return tasks
```

3 Constructing pseudo distributions

Pseudo distributions were chosen in accordance with the work of [2] where possible, which was based on the distribution of measured values in several databases. However, some of the distributions resulted in rather large differences in scale between values causing extreme stiffness in the system, which can lead to problems when doing the numerical simulations. Therefore, the mean was brought closer to 1 to avoid excessive stiffness in the numerical simulation. To compensate for this change, the distribution width was increased giving the pseudo distribution less weight during the parameter balancing phase.

Supplementary Table 1: Pseudo distributions when rates are unavailable in databases

Parameter	Distribution	Mean		Standard deviation	
		This study	Lubitz et al.	This study	Lubitz et al.
μ	Normal	-880	-880	680	680
k^V	Log-normal	10	10	2	1
k^M	Log-normal	0.01	0.1	1.2	1
k^I	Log-normal	0.1	0.1	2	1
k^A	Log-normal	0.1	0.1	2	1
c	Log-normal	0.1	0.1	2	1
u	Log-normal	1	0.0001	1.6	1.5
k^{eq}	Log-normal	1	1	2	1.5
k^{cat}	Log-normal	1	1	2	1.5
v^{max}	Log-normal	10	0.001	2	2
A	Normal	0	0	10	10
μ'	Normal	-880	-880	680	680

4 Compartmentalisation and regulation

Compartmentalization and metabolic regulation are essential in regulating metabolism [4,5], and thus, a metabolic modelling framework should be able to support these features. In Figure 4, we show how a randomly generated metabolic model can be regulated through these effects (see next section). For all simulations, the exact same model is used with the same parameter samples. The models were simulated for 400 s with a pulse in one of the metabolites after 200 s. Upon addition of compartments of different sizes to the basic model (Supplementary Figure 4A), we can see how the formation of a metabolite can be significantly slowed down (Supplementary Figure 4B), or how the addition of regulatory interactions can tightly regulate the steady state concentration of the same metabolite (Supplementary Figure 4C). Including both compartmentalization and metabolic regulation (Supplementary Figure 4D) leads to increased dynamics of the metabolite concentration, with decreased build up and lower variability. Additionally, we show in Supplementary Figure 5 that in the *L. lactis* model produced from our pipeline that altering regulatory mechanisms impacts the dynamics observed in simulated systems. In this instance, adding allosteric regulation has the greatest influence on system dynamics as glucose is not readily taken up, resulting in low F6P concentrations.

5 Generating Random Metabolic Networks

To generate random networks, a stoichiometry matrix and flux vector need to be created following equation (1) of the main text. To construct the stoichiometry matrix **S** we first generate metabolites, m, and the number of reactions, n, each m participates in is sampled from a discretised power law distribution $n(x) = x^{3/2}$ where $x \in [1, D_{max}]$. In our work $D_{max} = 8$. This means that a metabolite m could take part in 1 to D_{max} different reactions according to $n(x)$.

To determine how metabolites regulate one another, first a participation matrix **P** is constructed. Each element $P_{ij} = k \geq 1$ implies that k molecules of metabolite i, m_i, are involved in reaction n_j, either as a product, or a reactant.

$$\begin{aligned} n_j &: km_i \longrightarrow \ldots, \text{ or} \\ n_j &: \ldots \longrightarrow km_i. \end{aligned}$$

If $k_{ij} = 0$ then m_i is not involved in reaction n_j.

In order to highlight how this works, we present a brief example of a network containing two metabolites, m_1 and m_2, and two reactions, n_1 and n_2. The participation matrix generated reads

$$\mathbf{P} = \begin{pmatrix} 1 & 0 \\ 1 & 2 \end{pmatrix}.$$

Thus, we know that the reaction scheme is either

$$\begin{aligned} n_1 &: m_1 \longrightarrow \varnothing, \text{ or} \\ n_1 &: \varnothing \longrightarrow m_1; \\ n_2 &: m_1 \longrightarrow 2m_2, \text{ or} \\ n_2 &: 2m_2 \longrightarrow m_1, \text{ or} \\ n_2 &: m_1 + 2m_2 \longrightarrow \varnothing, \text{ or} \\ n_2 &: \varnothing \longrightarrow m_1 + 2m_2. \end{aligned} \tag{1}$$

The next step is to determine whether metabolite m_i is a reactant or a product. To do this we draw a random number $p_i \sim U(0, 1)$. If $p_i \leq 0.5$ then m_i is a reactant, else $p_i > 0.5$ and m_i is a product. To ensure that each reaction has a reactant and a product, metabolite m_{i+1} is a reactant if $p_{i+1} = 1 - p_i > 0.5$ and a product if $p_{i+1} = 1 - p_i < 0.5$. For all other metabolites, values of p_i are drawn randomly to determine whether it is a reactant or product.

For the sake of our example, let us say that for the first reaction, n_1, $p_1 = 0.4$ such that m_1 is a reactant of n_1. Since no other metabolites participate in this reaction it reads

$$n_1 : m_1 \longrightarrow \varnothing.$$

For our second reaction, n_2, $p_1 = 0.6$ such that m_1 is a product of n_1. Automatically, m_2 is forced to be a reactant of the reaction (remember these networks are randomly generated and have no biological relevance other than in network structure). Thus, from the possible reactions listed above, our reaction reads

$$n_2 : 2m_2 \longrightarrow m_1.$$

Finally, to obtain the stoichiometry matrix, we change the signs of all reactants in the participation matrix $\mathbf{P}$ to be negative, leading to the stoichiometry matrix $\mathbf{S}$

$$\mathbf{S} = \begin{pmatrix} -1 & 0 \\ 1 & -2 \end{pmatrix}.$$

Because of the inherent randomness used when generating the networks, it can happen that networks with unwanted properties — such as reactions without participants — are generated. Therefore a series of additional checks is performed and the procedure is repeated if there are any reactions without participants or when the stoichiometry of a reaction is above a set threshold.

In addition to the stoichiometry matrix, a localization array is randomly generated where each metabolite is assigned to a random choice of compartments, and a regulation matrix where metabolites can be assigned to either inhibit or activate a certain reaction with a certain mechanism according to equation (5) of the main text.

In order to complete equation (1) from the main text, a flux vector $\mathbf{v}$ is generated following equation (3) of the main text. This can be done in the same manner as in the main pipeline, with the parameter values being drawn from predefined distributions (Supplementary Table 2).

Finally, to generate *in silico* measurement errors on the parameters in the model, multiplicative noise drawn from a truncated normal distribution is multiplied to the true parameters. These parameter distributions can then be used in the parameter balancing method as a prior distribution.

Supplementary Table 2: Pseudo distributions for random metabolic networks

Parameter	Distribution	Mean	Standard deviation
μ	Normal	-10	1
k^V	Log-normal	1	2
k^M	Log-normal	0.1	1.2
k^I	Log-normal	0.1	1.2
k^A	Log-normal	0.1	1.2
c	Log-normal	0.1	2.0
u	Log-normal	1	1.6
k^{eq}	Log-normal	1	2
k^{cat}	Log-normal	1	2
v^{max}	Log-normal	1	2
A	Normal	0	2
μ'	Normal	-10	2

References

[1] W. Liebermeister, J. Uhlendorf, and E. Klipp. Modular rate laws for enzymatic reactions: thermodynamics, elasticities and implementation. *Bioinformatics*, 26:1528–1534, 2010.

[2] T. Lubitz, M. Schulz, E. Klipp, and W. Liebermeister. Parameter balancing in kinetic models of cell metabolism. *Journal of Physical Chemistry B*, 114:16298–16303, 2010.

[3] A. Flamholz, E. Noor, A. Bar-Even, and R. Milo. equilibrator - the biochemical thermodynamics calculator. *Nucleic Acids Research*, 40:D770–D775, 2012.

[4] S. R. Hackett, V. R. T. Zanotelli, W. Xu, J. Goya, J. O. Park, D. H. Perlman, P. A. Gibney, D. Botstein, J. D. Storey, and J. D. Rabinowitz. Systems-level analysis of mechanisms regulating yeast metabolic flux. *Science*, 354(6311):aaf2786–aaf2786, oct 2016.

[5] Annalisa Zecchin, Peter C. Stapor, Jermaine Goveia, and Peter Carmeliet. Metabolic pathway compartmentalization: an underappreciated opportunity? *Current Opinion in Biotechnology*, 34:73–81, aug 2015.

[6] R. S. Costa, A. Hartmann, P. Gaspar, A. R. Neves, and S. Vinga. An extended dynamic model of *lactococcus lactis* metabolism for mannitol and 2,3-butanediol production. *Molecular Biosystems*, 10:628–639, 2014.

Supplementary Figures

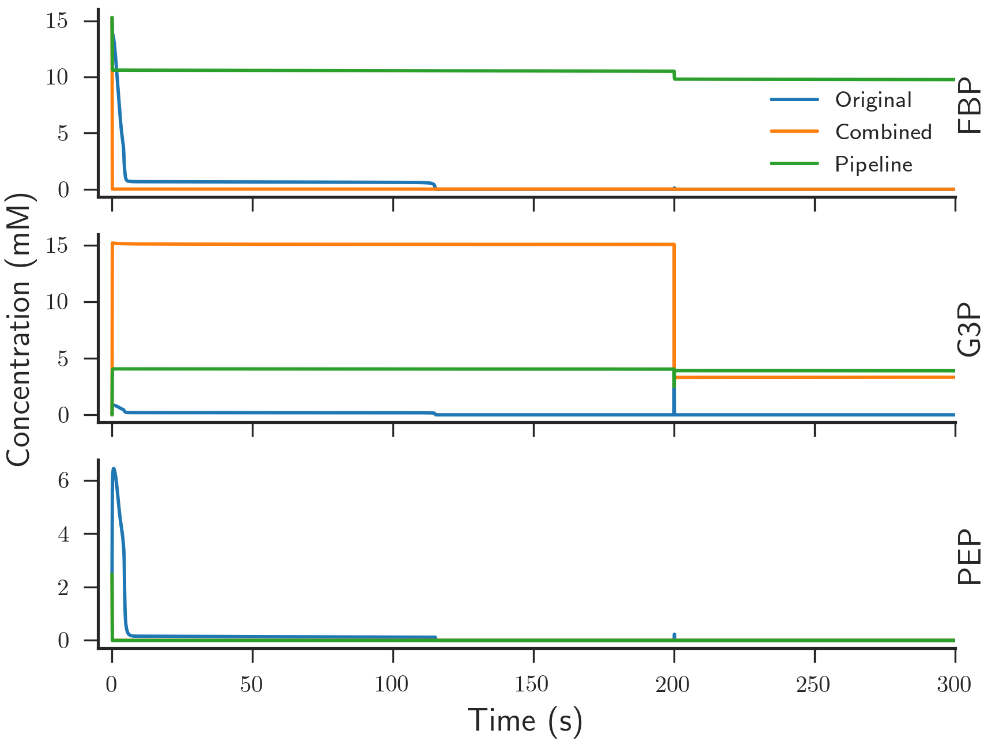

Supplementary Figure 1: System dynamics change depending on the input prior distributions. Simulations of (A) FBP, (B) G3P, and (C) PEP are altered given the data used to construct prior distributions. The blue line is the original model published by Costa *et al.* [6], the green line is the model created using the pipeline without the estimated parameters of Costa *et al.* [6] and the orange line is the model created using both the online databases and optimal values of Costa *et al.* [6]. It can be observed that for some metabolites the original simulation is close to the model resulting from the pipeline (PEP), but for others (G3P), the simulation is significantly different. Finally, some states such as FBP show a closer match when the values by Costa *et al.* [6] are taken as additional prior information.

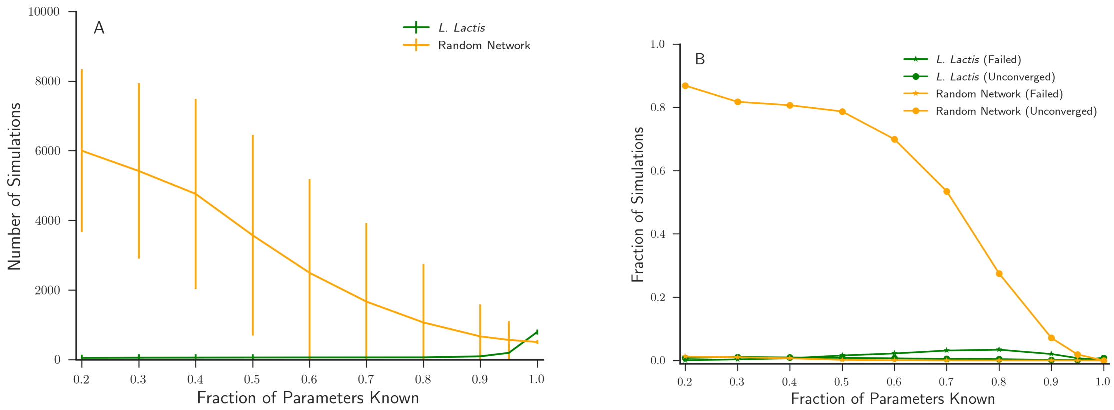

Supplementary Figure 2: Convergence and error rates in simulations of Costa and Random models. Given a percentage of known parameter values used as input into the pipeline, the posterior distribution is resampled until the mean error score between model simulations and the 'gold standard' dataset converges. (A) The mean number of samples before convergence of the mean error score is recorded. (B) The number of unconverged or failed simulations is recorded. Simulations can fail due to numerical issues when simulating the ODE system. When comparing the convergence of the *L. lactis* network compared to the random network, the convergence displays quite a different trend. The random network requires more simulations until convergence as would be expected at lower information. However, the mean number of samples before convergence of the *L. lactis* model quickly goes down with lower information. When we compare this to the results in Figure 5 and Additional File (Figure 3), one can see that the *L. lactis*, as opposed to the random network has several peaks. This might indicate that there are concentration states that regardless of the sampled parameters are more prevalent due to the network structure. These peaks, in turn, could cause the effect of quick convergence because of their prevalence when sampling the parameters.

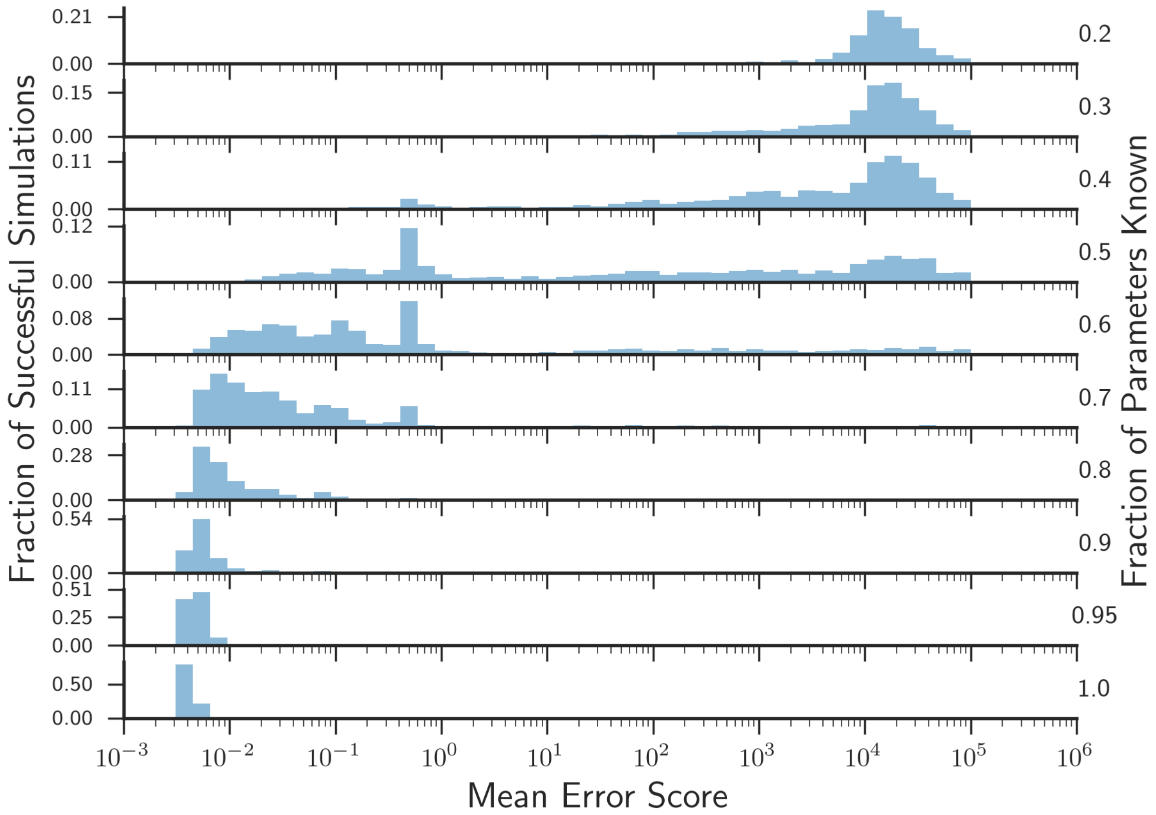

Supplementary Figure 3: Similar to Figure 5 in the main text, the robustness of the parameter balancing technique is tested for a random metabolic network. Given a percentage of known parameter values used as input into the pipeline, the posterior distribution is resampled until the mean error score between model simulations and the 'gold standard' dataset converges.

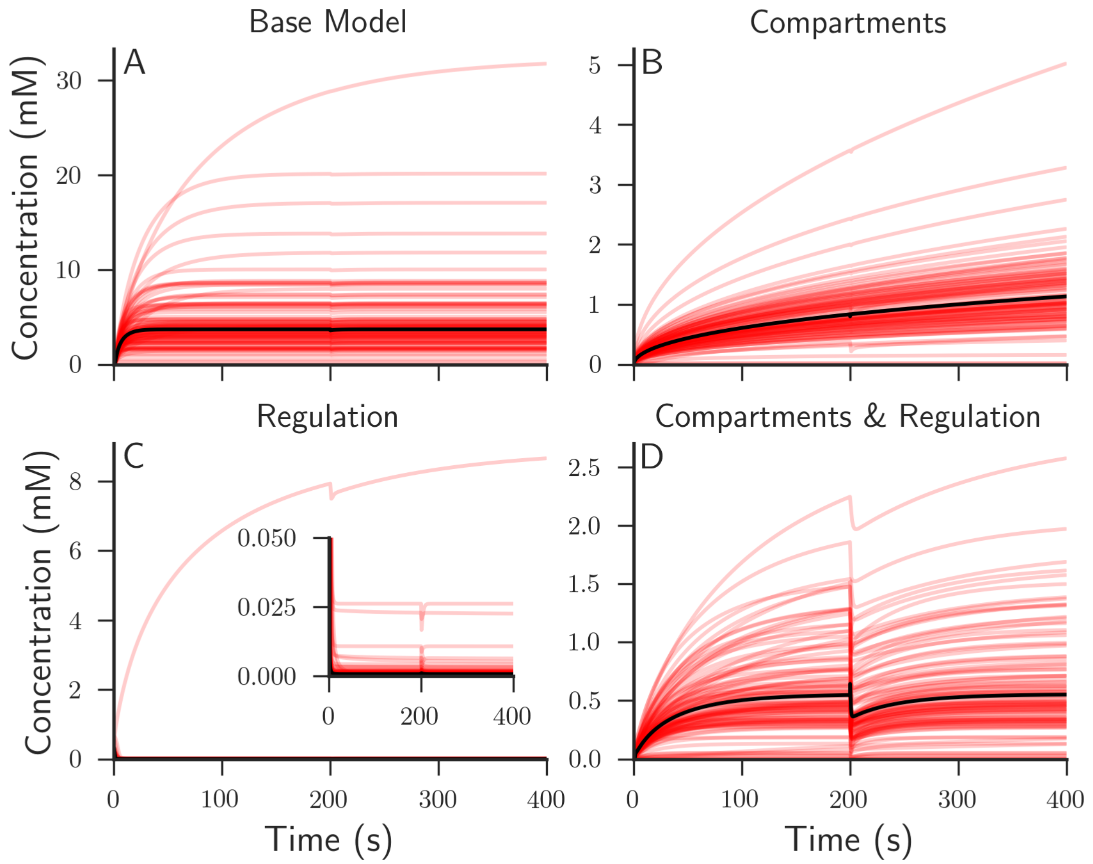

Supplementary Figure 4: Compartmentalisation and regulation of metabolic networks can be included in DMPy. Simulated dynamics of a random metabolic network when all cell compartments are equal in size (A), when one of the compartments is twice the size of the others (B), when randomized regulatory interactions are added (C) and both size differences and regulation is introduced (D). The inset in (C) shows the dynamics at low concentrations. All models were simulated for 400 s with a pulse after 200 s in one of the metabolites. Note that the random network and parameter samples are identical between simulations except for the aforementioned differences.

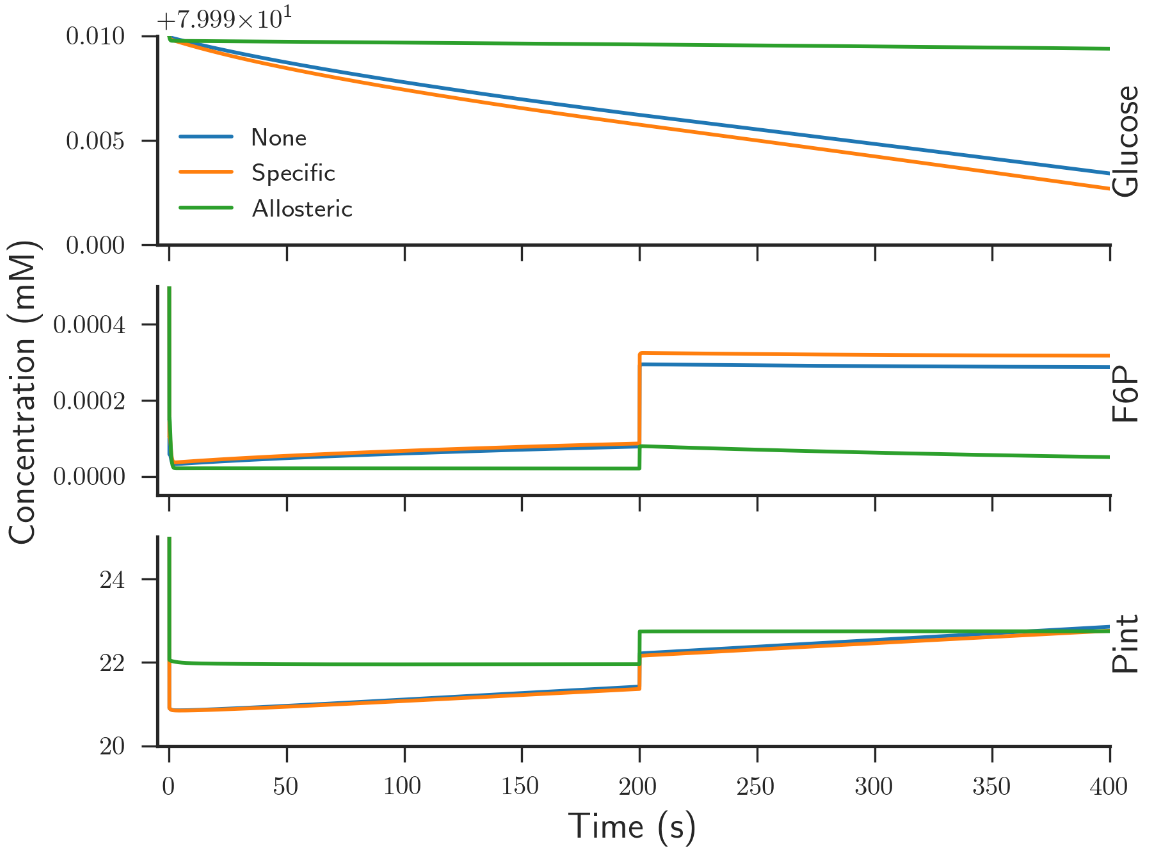

Supplementary Figure 5: System dynamics are affected by changes in metabolite regulation type. The *L. lactis* model produced by the pipeline is simulated when metabolites are regulated by allosteric regulation (green), specific regulation (orange) or unregulated (blue) - see equations (4) and (5) of the main text.
